# Superior adsorption of 3D nanoporous architectures for Ni(ii) ions adsorption using polyvinyl alcohol as cross-linking agent and adsorption conveyor

**DOI:** 10.1039/c8ra00113h

**Published:** 2018-02-19

**Authors:** Haibo Wang, Wei Wang, Yufen Zhao, Zhiwei Xu, Lei Chen, Lihuan Zhao, Xu Tian, Wanying Sun

**Affiliations:** State Key Laboratory of Separation Membranes and Membrane Processes, School of Textiles, Tianjin Polytechnic University Tianjin 300387 China xuzhiwei@tjpu.edu.cn +86 22 83955231 +86 22 83955231

## Abstract

In this study, we report a large-scale and low cost approach for the synthesis of three-dimensional (3D) polyvinyl alcohol/carbon nanotubes nanoporous architecture using self-assembly method. Polyvinyl alcohol, serving as a cross-linking agent and adsorption conveyor, could effectively interconnect carbon nanotubes sequentially and also effectively store Ni(ii) ions. An outstanding adsorption of 225.6 mg g^−1^ was achieved for 3D nanoporous structure, which was 18-fold more than that for carbon nanotube powders and much higher than that for other sorbents reported in literature. In addition, it was found that 3D nanoporous architectures remained intact after adsorption, which could recollect resources and avoid carbon nanotube leakage into water. Therefore, the designed 3D nanoporous architectures have a good potential application in environmental protection.

## Abstract 2

In this study, we report a large-scale and low cost approach for the synthesis of three-dimensional (3D) polyvinyl alcohol/carbon nanotubes nanoporous architecture using self-assembly method.

## Graphical abstract

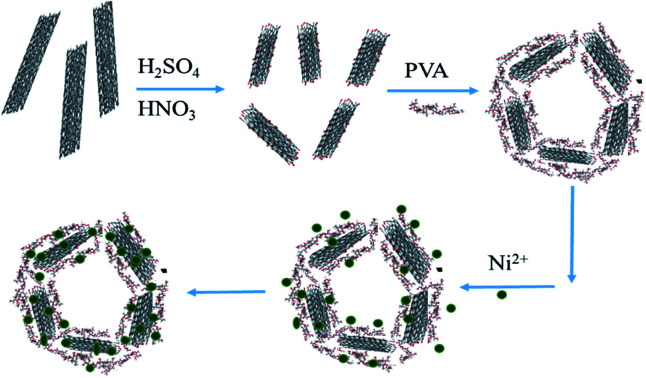

## 1. Introduction

It is well known that heavy metal pollution is a great global issue particularly in the developing countries. Among them, Ni(ii) ions, a common heavy metal, is widely distributed in the industrial applications such as batteries, electroplating and coinage. Moreover, excess amount of nickel is hazardous to the human body, particularly to the skin. Neurological disorder will occur on long term exposure to nickel, which will also lead to nose cancer and lung cancer.^1,2^ Hence, it is urgent and necessary to enrich and separate Ni(ii) ions from wastewater.

Several water treatment technologies, including co-precipitation, ion exchange, membrane filtration, and adsorption, have been investigated for removing Ni(ii) ions. Of these technologies, adsorption is the most promising technique due to its cost-effectiveness, ease of operation, and consistent performance.^3,4^ Due to large specific surface areas and hollow ability to be combined with metal oxides, carbon nanotubes (CNTs), which are one-dimensional substances, have great potential as superior adsorbents for removing Ni(ii) ions.^5^ However, this adsorbent has the disadvantages of low adsorption capacity or low removal efficiency because of severe aggregations occurring due to the strong van der Waals forces among them. Recently, nanoporous structures have attracted extensive attention owing to their unique characteristics such as high porosity, low density, and large specific surface area.^6–9^ Furthermore, traditional adsorption experiments require ultrasound or stirring to establish full contact of ions with the adsorbents, but Ni(ii) ions could not be spontaneously adsorbed this manner. In addition, after the adsorption process, it is difficult to completely remove or separate CNTs powder from treated water.^10^ It is necessary to design adsorbing materials, which are both good at storing ions and conducting them and also remain intact after adsorption experiment. Polyvinyl alcohol (PVA) is considered as an ideal carrier for adsorption applications due to its porous structure and good mechanical properties. In addition, PVA contains abundant hydroxyl groups on the surface, which can serve to adsorb heavy metal ions.^11–13^

Herein, we used PVA as a cross-linking agent and as an adsorption conveyor to engineer advanced three dimensional polyvinyl alcohol/CNTs nanoporous architectures (3DPCA) *via* self-assembly method. In addition, the 3DPCA as an adsorbent was applied to remove Ni(ii) ions from the aqueous solution. The factors that influence the adsorption performance of Ni(ii) ions, such as the contact time and initial concentration, were also evaluated.

## 2. Experimental

**Fig. 1:**
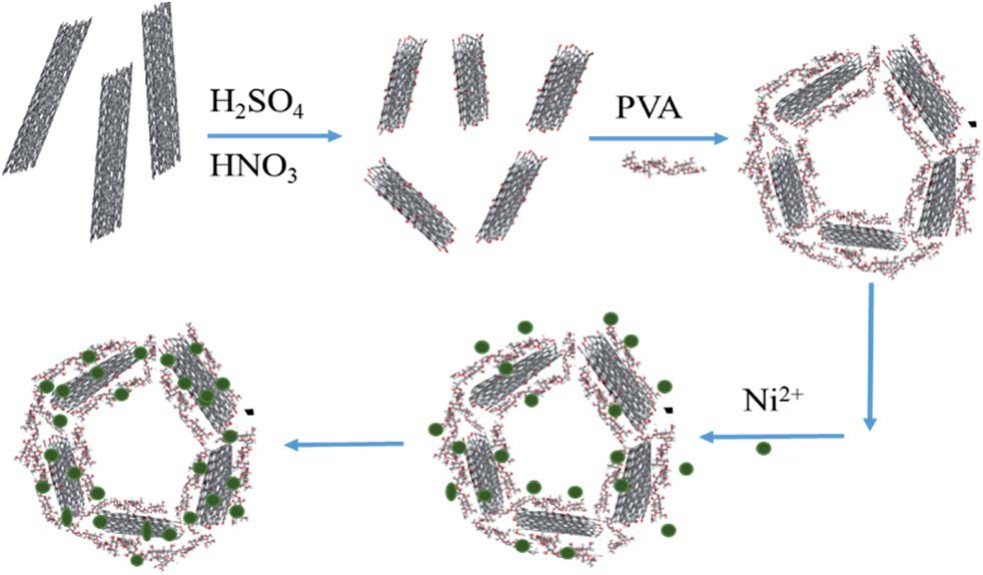
The schematic for the preparation of 3DPCA and adsorption of nickel ions from aqueous solution.

In order to introduce hydrophilic functional groups on the surface of CNTs, the CNTs were impregnated in H_2_SO_4_/HNO_3_ (3/1, v/v). PVA powder was completely dissolved in H_2_O at 95 °C. The homogeneous PVA solution was cooled to 50 °C and functional CNTs (FCNTs) suspension was added dropwise to the homogeneous PVA solution under stirring for 2 h. The as-prepared gelatin was freeze-dried for 48 h to obtain the 3D nanoporous FCNTs–PVA network. The Ni(ii) ion-solutions with different concentrations were prepared from nickel nitrate. Ni(ii) ions adsorption capacity was calculated from the following equation:
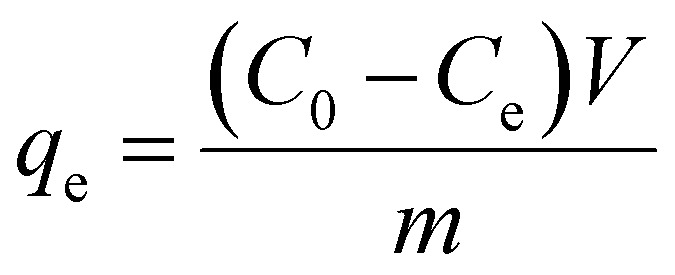
where *q*_e_ is the amount of Ni(ii) ions adsorbed (mg g^−1^), *C*_0_ is the initial concentration of arsenic in the solution (mg L^−1^), *C*_e_ is the equilibrium concentration of arsenic after adsorption (mg L^−1^), *V* is the volume of solution (L), and *m* is the mass of the adsorbents (g). The schematic was presented in Fig. 1.

## 3. Results and discussion

**Fig. 2:**
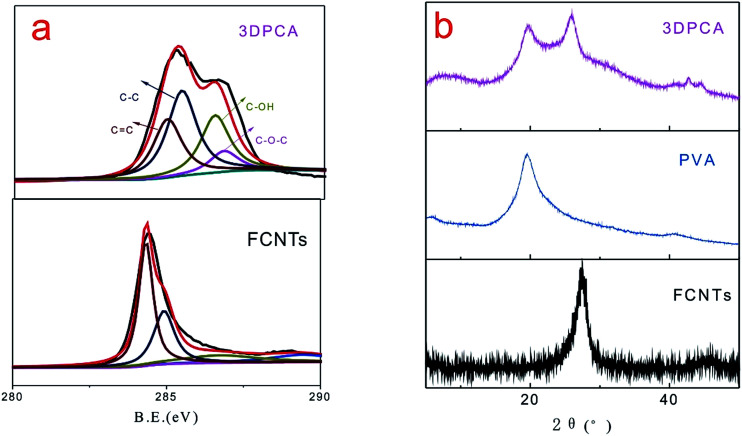
XPS spectra (a) and XRD patterns (b) of 3DPCA architectures.

XPS spectra of the obtained products are shown in Fig. 2a, which were recorded to further analyze the evidence of reaction mechanism. Fig. 2a shows the C 1s core-level XPS spectra of FCNTs and 3DPCA composites, which were fitted by a Gaussian–Lorentzian peak-shape to provide information about the functional groups. Four types of carbon peaks are centered at ∼284.5, ∼285.1, ∼286.6 and ∼287.3 eV, corresponding to C=C, C–C, C–OH and C–O–C groups, respectively. The C 1s signal was caused by the component of FCNTs and PVA chains. The C–OH and C–O–C absorption peaks of 3DPCA increased significantly, which originated from the presence of PVA. Moreover, these hydroxyl groups provided more active sites and serve to adsorb heavy metal ions.^14^ XRD is an efficient method used to evaluate crystalline structures of composites. XRD patterns of FCNTs and 3DPCA composites are shown in Fig. 2b. The diffraction peak of FCNTs observed at 26.6° originated from the (002) planes of graphitic carbon. The XRD curve of the 3DPCA may be considered as a simple combination of the XRD curves of both PVA and FCNTs. In presence of hydrogen bonding interactions between FCNTs and PVA, the intensity of PVA diffraction peak for 3DPCA is weaker than neat PVA.^15^

**Fig. 3:**
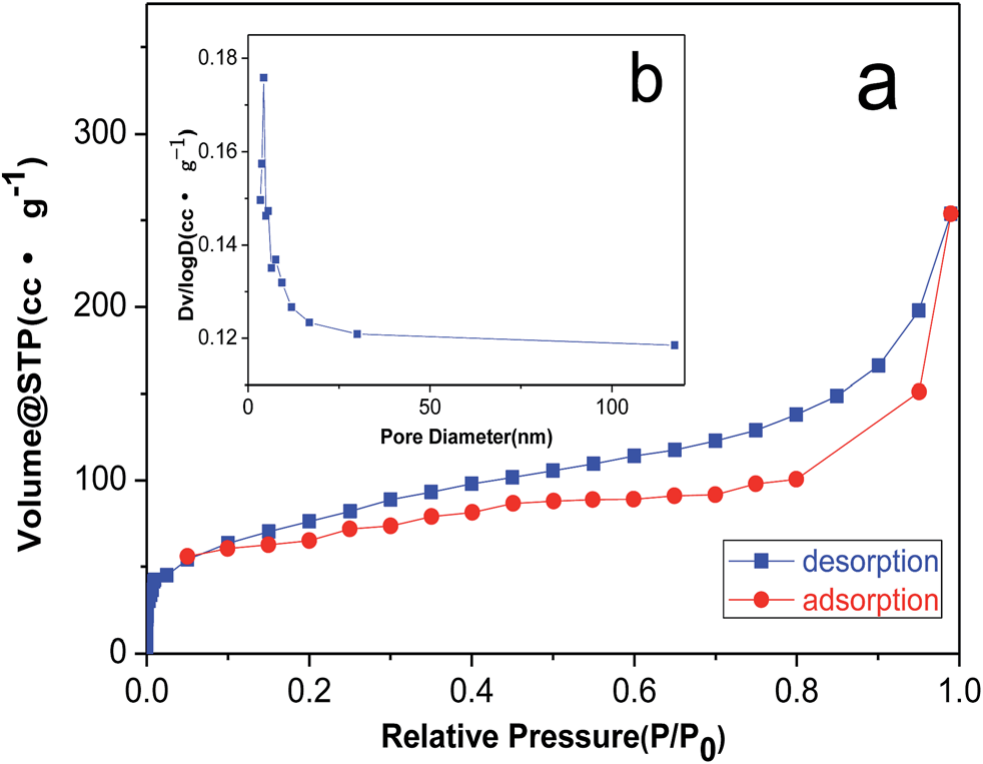
N_2_ adsorption–desorption isotherms (a) and the BJH pore size distribution plot (b) of 3DPCA.

In order to illustrate the pore structure of the material, we carried out N_2_ adsorption–desorption test. As shown in Fig. 3a, the isotherms for 3DPCA are ascribed to Barrett–Joyner–Halenda (BJH) type IV, indicating the mesoporous structure. The isotherm at the low relative pressure stage indicates the monolayer adsorption, which is followed by multilayer adsorption at the high relative pressure stage. The pore size distributions are calculated by the BJH adsorption method (Fig. 3b). The BJH cumulative adsorption pore volume demonstrates a narrow distribution of uniform mesopores in the range from 3.5 to 20 nm along with some mesopores and macropores.^14^

**Fig. 4:**
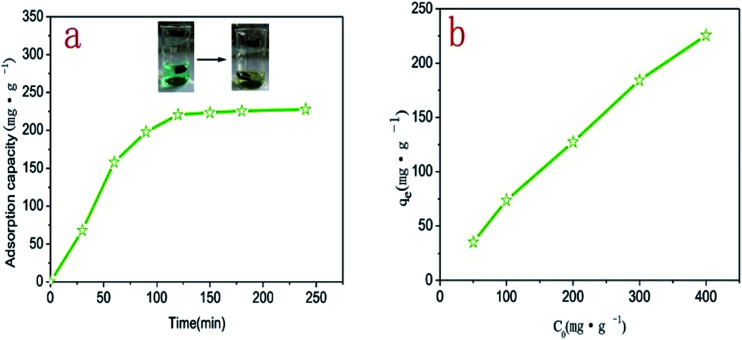
Effect of contact time and initial concentration on adsorption of Ni(ii) ions by 3DPCA.

The adsorption capacity of 3DPCA on Ni(ii) ion was studied at different contact times. As shown in Fig. 4a, with the extension of the contact time, the adsorption capacity increased rapidly and then reached dynamic equilibrium. The variation tendency could be explained by the reason that Ni(ii) ions were adsorbed from the external to internal nanoporous architectures and the adsorption capacity reached the dynamic equilibrium after adsorption saturation. In addition, the adsorption capacity reached 92% at 100 min relative to equilibrium adsorption capacity, indicating that the adsorption rate of 3DPCA for Ni(ii) ions was rapid.^16^ The initial concentration can significantly affect the adsorption efficiency of 3DPCA. As shown in Fig. 4b, the adsorption capacity on Ni(ii) ion increased gradually with the increase in the initial concentration. This phenomenon could be attributed to the fact that there were numerous adsorption vacancies in 3DPCA and the concentration gradient was produced between the solute and surface of the adsorbent. The gradient became larger when the initial concentration gradually increased, resulting in the enhancement of the adsorption capacity. The maximum adsorption capacity of the 3DPCA was achieved at 225.6 mg g^−1^ for the initial concentration of 400 mg g^−1^.

Adsorption process could be attributed to two major reasons. On one hand, conventionally used adsorbents have extremely complex surface chemical propertied, resulting in a wide variation in types of binding sites available to the solutes. On the other hand, there is participation of diverse driving forces between adsorbents and adsorbates, such as chemisorption, hydrogen bonding, electrostatic interaction, and complexation.^17^ In this paper, the synergistic effect between FCNTs and PVA for Ni(ii) adsorption should make 3DPCA an excellent adsorbent for Ni(ii) ions. First, the porous structure of PVA microsphere is conducive to the diffusion and adsorption of Ni(ii) ions. PVA is considered as an ideal conveyor for adsorption applications as it contains abundant hydrophilic groups (hydroxyl groups) on the surface. These hydroxyl groups could also serve to adsorb Ni(ii) ions and the existence of hydrophilic groups accelerates the adsorption process.^17,18^ Second, the FCNTs are favourable for Ni(ii) ions adsorption. The oxidation of CNTs surfaces is known to generate more oxygen containing functional groups and increase the ion-exchange capacity. More defects were brought into CNTs during the functional process and the Ni adsorption occurs on the defect sites favorably.^19,20^ Third, FCNTs are covalently bonded to PVA chains. PVA serving as cross-linking agent plays a fairly crucial role in constructing the porous network structure by efficiently interconnecting the FCNTs and keeping its integrity during the synthesis process. This nanoporous architecture has an interconnected 3D porous network with large surface-area-to-volume ratios, which provides more active sites for adsorbing and storing Ni(ii) ions.^21^ The 3DPCA nanoporous architecture, which combines both the advantages of FCNTs and PVA, should be an excellent adsorbent for Ni(ii) ions. Table 1 compares the adsorption properties of our as-prepared 3DPCA with those of other sorbents that have been reported in literature. It is worthwhile to mention that the 3DPCA nanoporous architecture possesses an excellent adsorption capability on Ni(ii) ions compared with other adsorbents.

**Table 1:** Comparison of adsorption capacities on Ni(ii) ion of some adsorbents

Adsorbents	Synthetic method	Adsorption capacity (mg g^−1^)	References
Ion-imprinted polymer	*Bulk polymerization*	86.3	4
GO–DPA	Ultrasonic-assisted	181	22
Hollow fibers	Mixed membrane	62.51	16
MWCNT	HNO_3_-treated	17.86	5
MgO nanosheets	Ultrasonic method	87	23
MgO nanosheets	Precursor calcination	185.5	24
PAO–AN	Chemical crosslinking	130	25
3DPCA	Self-assembly	225.6	This study

**Fig. 5:**
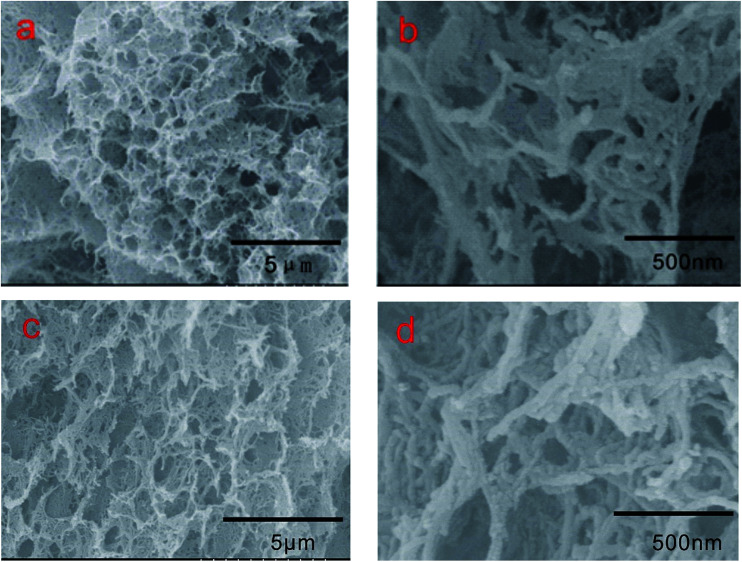
SEM images of 3DPCA architectures before (a and b) and after (c and d) adsorption.

Images of 3DPCA are shown in Fig. 5. In a typical synthesis procedure of 3DPCA, a black mixture of FCNTs and PVA was transformed to a self-assembled 3D nanoporous architecture after freeze-drying. Reinforcement with PVA polymer improved the strength and stability of the nanoporous architecture. As shown in Fig. 5(a and b), a freestanding 3D FCNTs and PVA skeleton was formed, which displayed a macro-porous structure with size of about 500 nm. As shown in Fig. 5a, the nanoporous architecture has an interconnected 3D porous network with randomly oriented structures. PVA serving as cross-linking agent plays a fairly crucial role in constructing the porous network structure by efficiently interconnecting the FCNTs and keeping its integrity during the synthesis process.^21^ In the absence of bundling, the nanotubes form a unique architecture with large surface-area-to-volume ratios, which are potentially useful for applications of adsorbing and storing heavy metal ions. The structure and stability of such nanoporous architecture after adsorption test was very important for its application. Fig. 5(c and d) display the existence of a continuous nanoporous network skeleton, which is well retained without any visible collapse after the adsorption experiment.

**Fig. 6:**
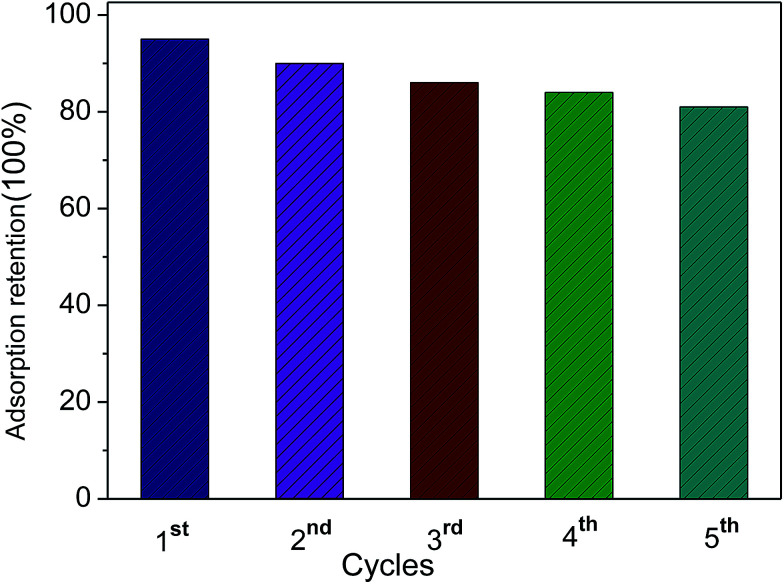
Five cycles of 3DPCA adsorbent for Ni(ii).

Furthermore, the durability of the adsorbing material is an important aspect that should be considered for practical industrial applications. The recycling of the 3DPCA was performed and the results are shown in Fig. 6. The adsorbent 3DPCA showed high recycling performance and maintained its adsorption capacity (∼83% of the first maximum) after 5 cycles of testing due to its robust interconnected network as illustrated in Fig. 5. No damage to nanoporous network was observed after this process as revealed by the SEM images, indicating the successful recycling and reusability of this material.

## 4. Conclusion

In this study, we used PVA as a cross-linking agent and as an adsorption conveyor to engineer advanced 3D polyvinyl alcohol/CNTs nanoporous architectures *via* self-assembly method. The material combining both the advantages of FCNTs and PVA exhibited excellent adsorbent performance for Ni(ii) ions. The maximum adsorption capacity was achieved at 225.6 mg g^−1^ for initial concentration of 400 mg g^−1^, which was much higher than other sorbents reported in literatures. In addition, it was found that the 3D nanoporous architectures with high loading of Ni(ii) ions remained intact after adsorption, which could be a good potential application for lithium ion battery, supercapacitor, and other fields.

## Conflicts of interest

There are no conflicts to declare.

## Supplementary Material
